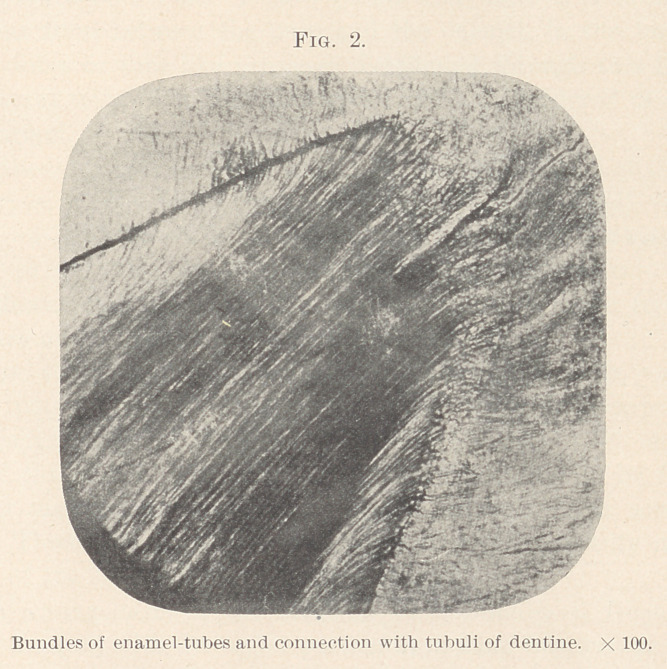# Reviews of Dental Literature

**Published:** 1904-06

**Authors:** 


### Reviews of Dental Literature.

Some Notes on the Enamel. By Douglas E. Caush, L.D.S.I.

Mr. President and Gentlemen,—In the paper I have the honor of readng before you to-night it is not my intention to deal with the question of the development of the enamel to any extent, but to bring before your notice a series of experiments I have carried out, and, as a result of these, to draw your attention to what appears to be some variations from the accepted theories of the microscopic structure of this tissue.

These experiments were commenced in the year 1888 for the purpose of finding, if possible, a reason for the staining of the enamel of human teeth by copper and other amalgams, the object being to stain the enamel, either from the outside or through the pulp; and at the same time to find out what portion of the tissue would take the stain, if any of it stained at all.

After a large number of experiments had been carried out, I found the following methods of staining were by far the most successful of all that were tried:

1. A number of teeth were placed in a five per cent. solution of chloride of gold for various times extending from two to ten days, after which the teeth were taken out of the stain, washed, and ground down until they were quite thin; these sections were then placed in distilled water, and exposed to the sunshine until they were almost black; they were then rubbed down between ground glass to get rid of the surface stain, and at the same time to make the sections thin enough for examination when they were mounted in balsam.

2. Teeth were placed in alcohol for some days, after which they were placed in an alcoholic stain of fuchsin for several weeks, in some cases even months; on taking them out of the stain the teeth were ground down in the usual manner and mounted for examination.

3. The teeth were placed in alcohol for a few days, then placed in a quantity of hot fuchsin stain and kept in a hot chamber for twenty-four hours, after which they were allowed to cool; when the teeth were ground down in the usual manner and mounted for examination. This method produced very good results, but the heat made the enamel much more brittle than the previous methods of staining.

After years of apparent failure I had almost given up hope of ever staining the enamel, when, a few months since, on examining one of the earlier prepared slides with a more powerful eye-piece, to my astonishment, and, I may say, delight, I found the enamel in the slides thus prepared had been much more thoroughly stained than at first appeared. This led to further experiments with eye-pieces of various powers. As a result of these experiments, I now use a compensating ocular $\times$ 18, with a comparatively low power objective (either eight or sixteen millimetres apochromatic), and find that I can see much more of the structure of the enamel, owing to the larger field, greater depth of focus, and the more perfect correction of color of the apochromatic lens, than when I use the microscope in the ordinary manner.

During the past six months a large number of sections (both longitudinal and transverse) have been prepared.

I have not confined myself to human teeth, but from *Primates* have made slides of the teeth of man and gorilla; *Carnivora,* fox and dog; *Ungulata,* cow, pig, and horse; *Rodents,* rats; all of which have been successfully stained by one of these processes.

In all cases the staining has been accomplished prior to the cutting of the sections of the teeth; and in no case have acids of any kind been used, either before or during the preparation of the teeth for the microscope. I have avoided the use of acids to prevent the possibility of any alteration of the tissues by decalcification.

Although for years I could see no staining of the enamel, at the same time I learnt much from the examination of these slides. Among other things, my attention was drawn to the fact that in every case those peculiar and constantly occurring portions of this tissue, known as the enamel spindles of Von Ebner, were always stained in the same manner as the tubuli of the dentine, whatever stain was used.

This led me to suppose, and I think proves, that there is a connection, or at least a means of communication, between the tubuli of the dentine and these spaces in the enamel. It also proves that the contents of these spaces are of an organic nature, uncalcified, probably protoplasmic in character, and possibly similar to the contents of the tubuli of dentine.

The staining also proves that there is direct communication between the pulp and these spindles. (Fig. 1.)

The constant and regular occurrence of these cavities shows that they are not pathological, as has been suggested by Mr. Charles Tomes, but normal, and probably play an important part in the life of the enamel. They vary very much in size, shape, and number, but in every case I think their existence is due to a check, or checks, in the calcification of the cells forming the enamel, during the process of development.

Besides the regular, uncalcified contents of these spaces, there are at times to be seen certain small glistening bodies, known as Römer's corpuscles. When they exist they are, I believe, composed of minute particles of calcified enamel. In all the experi-

FIG. 1.

Showing connection between enamel spindles and tubuli ; also enamel-tubes × 200.

FIG. 2.

Bundles of enamel-tubes and connection with tubuli of dentine. × 100.

ments made I have not been successful in staining them, and it is easy to understand how readily small, isolated, and calcified enamel cells may become surrounded by the non-calcified tissue in the early stages of development.

I do not think Von Ebner was correct when he suggested that these spindles contained air, and I am certain these cavities are not produced by the shrivelling up of the cement substance, as he suggests.

In those cases where air has been enclosed, if such a condition exists, it may be the result of having allowed the teeth to dry for some time prior to the sections being made from the teeth, for examination; even in these cases I have found that the action of the alcohol is to displace any air and to allow the stain to easily penetrate into the tissues; this is well demonstrated by the hot method of staining.

As far as my examination of the teeth of animals has gone, it is very unusual to find any pronounced cavities corresponding to the enamel spindles of man, though in some animals I have found what appears to be a modification of these spindles.

At the same time, in the teeth of all the animals from which sections have been made I have found that there is a means of communication between the pulp and the enamel, in the form of certain tubes or tube-like processes distributed through this tissue. So constant were they, that they could not be considered as other than normal.

As there appears to be no record of their existence, I shall, for the purpose of describing them, call them "enamel-tubes." There is apparently no sheath to these tubes, their position is between the enamel prisms; the calcified prisms forming their walls. They are, I think, produced by the non-calcification of the tissue (cement substance or otherwise) between the enamel prisms.

These tubes vary somewhat in size, according to their position in the tissue. Near the neck of the teeth they usually appear as short, separate, and distant tubes, but as we approach the cutting edge of the incisors and canines they are frequently to be found grouped together in bunches or bundles, as well as in separate tubes. These tubes also take this form very pronouncedly in the crowns of the bicuspids and molars. (Fig. 2.)

The arrangement of the tubes in these bundles is frequently that of a spiral, with the upper portion branching into two or more divisions. They sometimes radiate from their base like a fan, and as a consequence it is impossible to get the whole of the bundles into focus at once.

The connection between the pulp and these enamel-tubes is proved by the fact that they take the stain readily. For these tubes to be thus stained, the stain must pass through the pulp or pass in from the outside. Experiments show that it is easier to stain through the pulp than to stain direct through the enamel.

That these enamel-tubes are distinct canals or tubes containing uncalcified tissue may also be assumed from this staining, as well as from the fact that wherever there are cut ends they are always stained in the same manner as the cut ends of the tubuli of the dentine.

That they are not pathological is clear from the fact that they occur in the teeth of the gorilla, fox, dog, cow, pig, horse, rat, and alligator, as well as in human teeth. (All the animals I have at present examined.)

It is interesting at this point to trace the connection between the tubuli of the dentine and both the spindles and the enamel-tubes. Immediately under the enamel the tubuli of the dentine branch very much and frequently anastomose, thus forming a complete net-work of small tubes under the enamel margin.

In the pig the branching is more pronounced, and a rudimentary granular layer is seen. In the fox the granular layer is more pronounced, whilst in the cow there is a perfect granular layer, corresponding to, and continuous with the granular layer between the dentine and the cementum.

The tubuli of the dentine terminate in this layer on the one side, and the enamel-tubes often pass into it from the other side; thus making the ends of the tubuli and the granular layer the means of communication between the two tissues.

Besides the enamel spindles and tubes there appears to be a more or less complete net-work of uncalcified tissue passing between the prisms and capable of being stained. This continues until the outer portion of the enamel is reached, when we find a series of larger tubes passing from the outside towards the central portion of this tissue.

This method of staining enables us to follow the curvature of the prisms both in man and animals. In the incisors and canines this curvature is not very pronounced, except at or near the cutting edges, but in the bicuspids and molars the reverse is the case, for in both the curvature of the prisms are very pronounced; and I believe there is a very good reason for their existence.

We all know the ease with which the enamel can be cut along the line of fracture, where the prisms are straight and comparatively parallel, but try to do the same on the crown of a molar, and you will find the resistance is very great. The cause of the resistance is the curvature, and the crossing of the prisms: the reason for this curvature of the prisms is that they may overcome the strain put upon these organs during the process of mastication.

The enamel-tubes that appear on the outer portion of this tissue and pass inward are usually very regular, and generally run parallel to each other in human teeth, whilst in the cow, with these tubes are frequently to be found bundles of tubes similar to the bundles found on the inner surface of the tissue.

I said in the earlier portion of my paper that these tubes and spindles played a very important part in the life of the enamel. The following, I believe, are some of the functions of both enamel spindles and tubes: (1) To convey sensation from the outside of the enamel to the pulp, especially in cases of erosion, and sensitive enamel; (2) to allow for any expansion, or contraction that may take place in the enamel; (3) as a means of conveying nourishment to the enamel during the life of the pulp, as, unless there is some way of conveying nourishment to this tissue it must of necessity be a dead tissue from the time of its development.

I think it is the existence of these tubes that has misled Dr. Bödecker in the theory propounded by him in his work on the enamel. Trying to harmonize the theories expressed by him, with the views of the writers on this side of the water, I carefully prepared a number of sections according to his method, and certainly there was apparently much, at first sight, to substantiate the views expressed by him, but for this fact, he evidently had not taken into consideration the action of the acids used in his method of preparing the slides of enamel, and as a consequence drew wrong conclusions from the sections thus prepared.

That which Dr. Bödecker designated the enamel-fibre is probably that portion of the enamel which has taken the stain in the slides I have prepared, whilst the other portion (the reticulum) described by him I do not think exists in the enamel under ordinary conditions; it has been artificially produced, as the result of the partial decalcification of the enamel prisms by the acid used in preparing the sections.

There are two other portions of this tissue to which I wish to call your attention: (1) the brown striæ of Retzius; (2) Schreger's lines.

(1) The brown striæ are, I believe, produced as a result of a difference between the refractive index of the enamel prisms and the tubes between the prisms.

Mr. Leon Williams, when referring to the brown striæ, says, "These markings are due to pigmentation." This, I think, is not quite correct, for in those sections where the enamel is very thin the prism between the tubes is as free from color or pigment as it is in any other portion of the tissue; whilst the tubes themselves are well colored and very pronounced. In thicker sections there is the appearance of pigmentation. This is due to the fact that where there is more than one layer of cells the tubes are not directly under each other, and the difference between the refractive index of the tubes and prisms produces the results seen.

In transverse sections stained in this manner the striæ are well shown, and it is not at all unusual to find a small portion of more perfectly calcified tissue on either or both sides of these markings, which by contrast makes the striæ appear more pronounced. May not these more perfectly calcified portions indicate that there were times of rest as well as times of activity in the development preceding, or following, the formation of the striæ?

In some sections, owing to the angle at which they are cut, the ends of these tubes form a continuous line of colored dots along the line of the striæ.

These brown striæ are not confined to human teeth, as I have found them in the teeth of some of the animals examined.

(2) Lines of Shreger. In these lines, though the cause is the same as in the brown striæ of Retzius, the appearance is quite different, and this is accounted for by the fact that in the brown striæ the stained tubes run parallel to each other, and those forming one of the sections of the brown striæ are very much in the same plane. This is not so in those markings known as the lines of Schreger. The tubes and prisms in Shreger's lines are very much curved, and the optical effect of this curvature is to produce the peculiar cloud-like markings characteristic of these lines. But if, whilst examining these lines under the microscope, the focussing is altered, it is possible to follow the course of these lines, and the the cloud-like effect is the result of the softening produced by the curvature of both prisms and the tubes.

As I said at the beginning of my paper, it is not my intention to deal to any extent with the development of this tissue. That subject is so vast, that were I desirous of doing so, the time at my disposal would be far too short. At the same time, as far as my study of this fascinating subject has gone, I think the theory expressed by Mr. Charles Tomes is more correct than that of Mr. Leon Williams.

If we accept Mr. Tomes's theory, that the enamel is produced as the result of the direct calcification of the enamel-cells, all the modifications I have shown to-night are easily explained, and their development can be readily understood. If, on the other hand, we accept the theories propounded by Mr. Leon Williams on the development of this tissue, so clearly and ably expressed in that splendid monograph of his on "The Enamel," these variations cannot exist, or, if they do exist, then there is much Mr. Williams has left unaccounted for.

To illustrate one point. Mr. Williams says, whilst discussing the theories of Von Ebner on the formation of the brown striæ, "that the idea is a mistaken one, first, because these supposed canals have no existence; and, secondly, because the ground-off ends of the enamel prisms do not appear except when the section of the enamel is ground at a certain angle."

In the face of such pronounced differences, pronounced with regard to the development of this tissue, what a splendid work our Society would accomplish if it were possible for them to appoint a committee to investigate this important matter. Should our Council see their way clear to carry out such a scheme, I shall be delighted to lend my slides to them for the purpose of examination and comparison with other slides to try and find out what is true in this matter.

In bringing this, my imperfect, and I am afraid somewhat rambling, paper to a conclusion, I believe your attention has been directed to the portion of the enamel that is stained by copper and other amalgams.—*Transactions of the Odontological Society of Great Britain.*

On Electric Sterilization of Root-Canals. By Dr. W. D. Miller, Berlin, Germany.

Mr. President and Gentlemen,—About two hours ago I was absolutely innocent of any design of occupying your attention this evening, but I happened to ask Mr. Mummery, when dining with him, whether he had ever made use of electric sterilization in the treatment of pulp-canals. He said he had not, and immediately pounced upon me for remarks on the question this evening. The subject of electro-sterilization of pulp-canals and diseased condition of teeth and the surroundings is by no means new. You are no doubt acquainted with the work done some four or five years ago by Zieler, a Russian dentist, residing in Würzburg. He made a number of experiments, using an apparatus very similar to that which we use for cataphoresis, conducting a current of about three milliampères through the root-canals for ten minutes; he reported most favorable results.

Slightly previous to this a dentist in Vienna, Dr. Brauer, also published results which tallied with those of Zieler. The matter, however, rested as it was until about six months ago, when one of my colleagues at the Dental Institute of the University of Berlin, Dr. Hoffendahl, came to me and reported enthusiastically about the results he had obtained by the use of an electric current in the treatment of root-canals. He cited a number of cases of chronic abscesses and fistulæ which had been treated in the ordinary way without any result being obtained, and other cases in which the teeth remained comfortable as long as no filling was inserted, but the moment the filling was inserted the teeth began to hurt. By treating those canals with the electric current he was able to reduce the inflammation, and repeatedly he noticed that the suppuration stopped entirely after two or three treatments, and that the teeth could be filled without any discomfort. That brought the matter again to my attention, and I decided to look into it a little closer, and make some bacteriological experiments. I repeated the experiments, I think, in all some twelve or fifteen times, taking freshly extracted putrid teeth, conducting an electric current of one and one-half to two milliampères through the canals for ten minutes, and, in order to bring about conditions as similar as possible to those of the mouth, I took the head of a calf, spread the jaws open, and implanted a tooth containing a putrid pulp in the anterior part of the upper jaw. The positive pole of the battery was inserted in the root-canal and the negative applied to the anterior part of the lower jaw. That gave a current passing through the whole head of the calf. In dealing with a patient we put the positive pole in the canal of the tooth we wish to treat, and the patient takes the negative pole in his hand, so that the current passes through two or three feet of tissue. We were able to determine, in nearly all cases, that the bacteria were completely destroyed by the current of one and one-half to two milliampères passing through the root-canal for a space of ten minutes. Experiments were made by testing the contents of the canal before beginning the experiment for the presence of bacteria, and also again after five minutes and after ten minutes, and in the majority of cases, after ten minutes, there were no living bacteria to be found, and usually after five minutes the number was very materially reduced. I also made experiments with *Staphylococcus pyogenes aureus* by infecting the canal with this bacterium, and found the same results. In order to determine whether the electric current would have the effect of sterilizing the contents of abscesses, I constructed a little apparatus consisting of a glass tube broadened at one end and drawn out at the other to a fine point, somewhat like a funnel. The positive pole was placed at the narrow end of the glass, representing the root of the tooth, and the broad end was supposed to represent the abscess. This was fixed into a plate of thick cardboard, and filled with a solution of bouillon that had been infected with Prodigiosus. The current was conducted through this solution, the negative pole being on the opposite end of the cardboard, after the whole had been impregnated with chloride of sodium, so as to make a conductor and get about the same conductivity as we have in the tissues of the human body. After ten minutes no diminution in the number of bacteria had taken place; but after twenty minutes the number had been reduced about half, and in thirty minutes, with a current of one and one-half to two milliampères, the number had been reduced to about one-fifth of the original number on starting. So that we see there is a certain action upon bacteria even in larger cavities. In this case I used a tube about ten to twelve millimetres in diameter, which represents perhaps something larger than the abscesses with which we usually have to deal.

The canal is kept moist with a physiological solution of common salt during the operation, and the sterilizing action is not due to the direct action of the electric current, but to that of the products of electrolysis, in particular to that of chlorine.

The bacteriological results I obtained were, on the whole, very favorable, confirming those obtained by Brauer and Zieler, and showing beyond a doubt that we are in a position to diminish, if not completely destroy, the bacteria by the use of the electric current. If that should turn out to be applicable in practice, it would be of immense value to us. We all know how difficult it is to treat the buccal roots of upper molars, or the mesial roots of the lower molars, and how impossible it is sometimes to penetrate them with the finest broaches. And if we are able to put an electric current, something as subtle, if not more subtle than the bacteria themselves, on their track and to penetrate the finest canals, exerting a destructive effect on the bacteria, it would be a most beneficial and wonderful advancement in dental surgery. The results which I have obtained in the application of this method in the dental clinic have been, on the whole, very favorable. In one case in particular we had to treat an upper central incisor, which for five weeks had been under the direction of one of the assistants, who had never been able to close it up without producing pain on the following day. Dr. Hoffendahl gave it one treatment, and sealed the tooth up, and the patient came next day and had not had the slightest trace of pain.

Nearly all the cases we have treated have turned out very favorably. In one case, however, the day following the treatment a large swelling on the cheek occurred. In this case we had used the electric current without cleaning out the root-canal at all, and it occurred to me afterwards that possibly the electric current had had the effect of conducting the ptomaines in the root-canal through into the tissue and brought about infection. I wrote to Zieler, who is now in Hamburg, and asked him whether he had given up the method. He said he had not given it up, but owing to his being continually moving about he had been unable to continue his work, but he was thoroughly convinced of the great utility of the method. I asked him if he had ever had a case of swelling occur after application of the current, and he said that he had not. I advise any one using the method to first clean out the root-canal as far as possible, or at least the easily accessible portions, before applying the electric current, because it seems to me there is a possibility of ptomaines present in the pulp being conducted into the tissue.

We are, as I say, at present at work on this method, and I have not given any particular attention to the battery. I am using a chromic acid battery of thirty to forty cells with rheostat, provided me by Dr. Hoffendahl. We begin with a very low current, a fraction of a milliampère, and gradually increase the current to one and one-half or two milliampères, and let it pass through for ten minutes. The patient, in the majority of cases, experiences no inconvenience whatever. Sometimes they say they have a slight sensation, not amounting to pain. After ten minutes the current is removed, and the tooth may be filled. The experiments, however, are only in progress at present, and I would prefer that you should not make use of it until you receive the result of our further experiments, because methods of this kind cannot be tested too thoroughly. New methods are often introduced, and give wonderful results at the beginning; but later on we find the results were only fictitious, and that the method has to be given up. I will take the liberty of keeping you informed of what further results we may obtain, and, if they continue to be as favorable as they have been heretofore, I shall be able to advise you to make experiments in the same line.

The President, while he could not help admiring Dr. Miller's persistence in his careful experimentation, felt still more admiration for the interesting and frank way in which he had stated the unsuccessful experiment, and for the warning he extended to dental surgeons not to play with the method until a little more information had been received from head-quarters. The most interesting point was that of the uncleaned canal. At first sight it seemed that the method would afford a delightful way out of the difficulties of dealing with those irregular canals which refused to be treated by ordinary methods of bristles, and so forth. He had felt that perhaps the current might be able to go where the bristles would not, and at least as far as the bacteria could permeate. But apparently it was necessary to be very careful, and await a further communication from Dr. Miller at some later date.—*Transactions of the Odontological Society of Great Britain.*

Starting-Pits: Their Use and Abuse. By S. H. Guilford, D.D.S., Ph.D.

The advisability or inadvisability of the use of starting-pits in the placing of gold fillings has long since ceased to be a subject for discussion either in the journals or the societies. The line of division between those who believe in their value or those who do not has been pretty sharply drawn, and each side seems satisfied with its respective method.

The subject would not now be revived in this journal were it not for the fact that a few examining boards have recently taken occasion to express strong disapproval of their employment when recent graduates were doing their practical work before them.

That small pits or depressions as aids in the starting of gold fillings were in very general use a quarter of a century ago, and that their employment has very greatly decreased since then, is certainly true; but that quite a number of practitioners still find them serviceable in certain cases is also true.

The decline in the use is most probably due to two causes,—first, their too general and often improper employment; and second, the advancement made in the methods of shaping cavities and inserting fillings.

They were called "retaining pits" originally, and this misnomer probably caused much of the prejudice which grew up against them. In many cases they were employed for purposes of retention, because the practitioner had a wholesome dread of his fillings dropping out and desired to take every precaution against such an unfortunate mishap.

The shaping of cavities to give them a proper retentive form was not so well understood in those days as it is now, but even when improvement came in this direction the "starting-pit," as it came to be called, continued to be used for the more secure anchorage of the first pieces of gold placed in a cavity.

Through ignorance and lack of experience their use often led to abuse, and so they fell more and more into disfavor. As used by Webb and other renowned operators, the starting-pit was made with a very small drill, and besides being very shallow, it was formed in the dentine very near to the enamel.

Others, less skilful or less prudent, made them large and deep, and often located them very near to the pulp.

There could be but one result in such cases,—namely, the death of the pulp with its unfortunate consequences. That a reaction should follow such practice was not only natural but inevitable.

In the more recent methods of shaping cavities, as advocated by Black and others, the cavity, if a compound one, is largely flattened at its cervical aspect, thus forming angles with the lateral walls. These angles in the dentine are intensified for the double purpose of more readily starting the filling and for its more secure retention.

In cavities of this class very few operators, if any, make use of a starting-pit, because the newer method does away with its necessity. In simple cavities on the approximal surfaces of teeth, however, especially the incisors and cuspids, a small, shallow starting-pit, properly located so as to avoid all danger to the pulp through thermal changes, has certain advantages, as many believe.

Each practitioner is naturally governed by his experience in the use or non-use of this aid to filling, but with the student who has had little opportunity to gain experience, and whose skill in overcoming difficulties has not had time to develop, the case is different.

Probably nothing proves so discouraging to the student in his efforts to acquire the art of properly introducing a filling as to have it shift its position when once started, or possibly come out entirely after it is completed, because its position has changed without his knowledge.

Teachers often find it necessary to advise and encourage their students to take advantage of certain aids which will assist them in their work and inspire confidence, knowing full well that as they advance in knowledge and experience many of these aids will be laid aside.

Thus we find students adjusting the rubber dam in all or nearly all cases where a gold filling is to be inserted and encircling each included tooth with a ligature.

Later, experience teaches them that in many simple cases both dam and ligatures may be discarded in favor of the napkin, to the advantage of the patient and themselves.

Excessive undercutting of cavities and the over-annealing of gold are excesses often practised by students in their overanxiety to secure a successful result.

Nearly all good points of any operative procedure are likely to be carried beyond the bounds of advisability by the inexperienced, but all these matters right themselves in time.

Students are usually taught correctly, and while the advantages of certain methods are pointed out, it is also made plain to them that the same methods carried to excess may result in injury instead of good.

We cannot expect a recent graduate to be as proficient as he is likely to become after years of practice.—*The Stomatologist.*

## Figures and Tables

**Fig. 1. f1:**
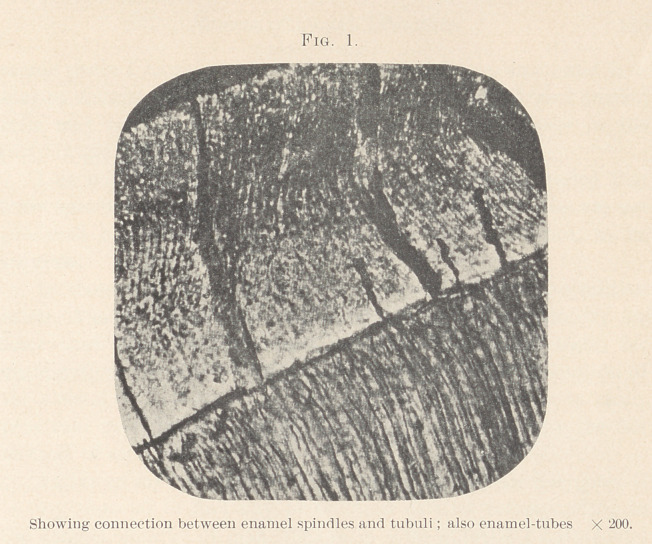


**Fig. 2. f2:**